# Bioimpedance Resistance Indices and Cell Membrane Capacitance Used to Assess Disease Status and Cell Membrane Integrity in Children with Nephrotic Syndrome

**DOI:** 10.1155/2019/4274856

**Published:** 2019-05-09

**Authors:** Steven Brantlov, Lars Jødal, René Frydensbjerg Andersen, Aksel Lange, Søren Rittig, Leigh C. Ward

**Affiliations:** ^1^Department of Procurement & Clinical Engineering, Aarhus University Hospital, Denmark; ^2^Department of Nuclear Medicine, Aalborg University Hospital, Denmark; ^3^Department of Paediatrics and Adolescent Medicine, Aarhus University Hospital, Denmark; ^4^School of Chemistry and Molecular Biosciences, The University of Queensland, Brisbane, Australia

## Abstract

**Background:**

Accumulation of extracellular water (ECW) is a major clinical manifestation of nephrotic syndrome (NS) in children. Bioimpedance spectroscopy (BIS) is a simple, noninvasive technique that reflects body water volumes. BIS can further measure cell membrane capacitance (C_M_), which may be altered in NS. The aims of the study were to explore how BIS measurements could reflect disease status in NS, while avoiding prediction equations which are often only validated in adult populations.

**Methods:**

The study involved 8 children (2-10 years) with active NS (ANS group), 5 of which were also studied at NS remission (NSR group), as well as 38 healthy children of similar age (HC group). BIS measurements determined resistances R_INF_, R_E_, and R_I_ (reflecting total body water, extracellular water, and intracellular water) and C_M_. Also resistance indices based on height (H) were considered, RI = H^2^/R.

**Results:**

It was found that R_E_ and R_INF_ were significantly lower in the ANS group than in both NSR and HC groups (p < 0.001). Corresponding resistance indices were significantly higher in the ANS group than in the NSR (p < 0.01) and the HC (p < 0.05) groups, in accordance with elevated water volumes in NS patients. Indices of intracellular water were not significantly different between groups. C_M_ was significantly lower in the ANS group than in NSR and HC groups (p < 0.05).

**Conclusion:**

BIS could distinguish children with active NS from well-treated and healthy children. Studies with more children are warranted.

## 1. Introduction

Nephrotic syndrome (NS) is a kidney disease characterized by the massive leakage of proteins into the urine, with consequent hypoalbuminemia and oedema formation [1], increasing the risk of complications and prolonged hospitalization [2]. Clinicians typically use body weight as a measure of the volume status in NS patients. However, this approach does not provide information about the different body water compartments; furthermore, using body weight as an indicator of volume status is only reliable for time periods short enough that changes in adipose tissue and muscle mass are nonsignificant [3].

Availability of a simple, inexpensive, and harmless method for routine assessment of volume status could provide new and clinically useful information to clinicians in the treatment of children with NS. Bioelectrical impedance spectroscopy (BIS) may prove to be such a method. BIS is noninvasive, harmless, quick, simple, and an inexpensive method that can be performed with a portable instrument. These features make it suitable for routine use.

Bioelectrical impedance is the opposition (impedance) of the body tissue against the flow of electrical current. The bioimpedance depends on the body composition, especially the distribution of water and nonwater in the body. Scales claiming to measure not just your weight but also your body composition make use of bioimpedance; however, most of these devices measure only at a single frequency. BIS measures bioimpedance at a wide range of frequencies, giving more complete and more reliable information.

BIS has recently been used to demonstrate increased volumes of extracellular water (ECW) in NS patients compared to controls [4]. As well as being able to provide information about the volume of the different body water compartments, BIS also estimates cell membrane capacitance (C_M_), which has been suggested as the parameter directly linked to the cell membrane function [5]; low C_M_ has been linked to disease in children [6]. To our knowledge, however, C_M_ has not been studied before in relation to NS patients.

While bioimpedance-based approaches for quantitative estimation of water volumes exist, these methods are based on prediction equations, which are prone to bias and imprecision. When only a single frequency is measured, the prediction equations are typically simple and purely empirical [7]. This makes the equations sensitive to the choice of population (e.g., adults but not children) as well as the precision of the reference method. In the multiple-frequency approach of BIS, empirical data are incorporated into equations derived from a biophysical model of body water compartments, their volumes, and inherent electrical resistivity [5]. However, even equations involving analytical derivations will depend on their assumptions of, e.g., homogeneity of the body. Accordingly, such prediction equations have been found inaccurate and population specific [8–10] in paediatric populations.

An alternative approach to using prediction equations may be the use of BIS resistance parameters, either as the resistances (R) or as the so-called resistance indices (RI) [11–13]. The resistances are calculated directly from the measured BIS data, while the resistance indices are calculated from the resistances and the subject's height.

**Figure 1:**
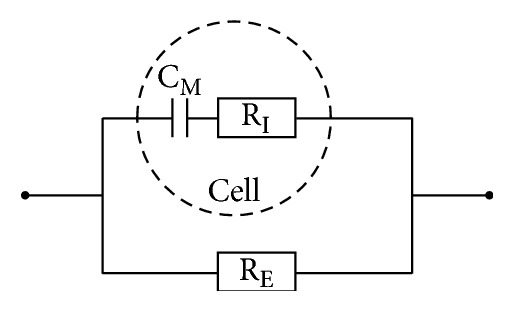
Model representing the electrical properties of body tissue [21]. R_E_ corresponds to the electrical resistance offered by the extracellular water (ECW) and likewise R_I_ from the intracellular water (ICW). C_M_ represents (at the body level) cell membrane capacitance of the double-layered cell membrane, i.e., the cell's ability to uphold a charge difference between the two sides of the membrane. The BIS device applies an alternating current (AC) and measures the combined impedance. Due to C_M_, the impedance will depend on the frequency of the electrical current, because high-frequency currents pass the cell membrane with less opposition than low-frequency currents. R_E_ and R_I_ are measured in Ohm (Ω), while C_M_ is measured in nanofarad (nF).

Briefly, to the BIS apparatus, the body can be represented by the electrical circuit shown in Figure 1. The measured parameters comprise the resistances R_E_, R_I_, and R_INF_, representing the electrical resistances of ECW, intracellular water (ICW), and total body water (TBW = ECW + ICW), respectively. For more details on BIS theory, see [5, 14]. Given the resistances R_E_, R_I_, and R_INF_, the corresponding resistance indices RI_E_, RI_I_, and RI_INF_ can be calculated, which (unlike the resistances) are roughly proportional to the water volumes [15].

Generally, there is a growing interest from the scientific community in the use of raw impedance data as indices of body water volume, especially in patients with altered body water distribution [16, 17]. These indices may be less intuitive than absolute body water volumes in L, but avoid invoking the various assumptions that underpin the prediction equations.

The aims of the present study were to investigate how BIS measurements can reflect disease status in NS, including both resistances (R_E_, R_I_, and R_INF_), corresponding resistance indices, and capacitance C_M_. For an overview of parameters and abbreviations, see Table 1.

## 2. Material and Methods

Standardized testing and reporting procedures were followed as far as possible [15, 18]. The datasets produced during and/or analysed during the present study are available from the corresponding author on reasonable request.

### 2.1. Subjects

Eight children (7 boys, 1 girl, age range: 2-10 years) with active NS (ANS group) were enrolled at the Department of Paediatrics and Adolescent Medicine, Aarhus University Hospital, Denmark. Inclusion criteria were the presence of NS defined by proteinuria >40 mg/m^2^/day, plasma albumin <25 g/L, oedema, and hyperlipidemia. Exclusion criteria were low plasma levels of C3-complement, postinfectious glomerulonephritis, and vasculitis such as Henoch-Schönlein nephritis of specific glomerulonephritis as, for example, dense deposit disease. Five of the ANS patients (ANS*∗* group) were also restudied on remission (NSR group). Remission was defined as urinary dipstick negative for protein on three consecutive days. Blood samples, blood pressures, and impedance measurements were collected in the ANS patients before treatment with prednisolone and diuretics was initiated.

For comparison, impedance measurements were also made in 38 healthy control children (HC group) (23 boys, 15 girls, age range: 2-10 years). These controls were taken from a previously published dataset [20].

### 2.2. Ethics

Informed consent was obtained from the subjects' parents or legal guardians before study enrolment. The study was performed in accordance with the Helsinki Declaration and was approved by the Central Denmark Region Committees on Health Research Ethics (case number: 1-10-72-17-12).

### 2.3. Patient Preparation

Participants were not fasting but had refrained from intense physical exercise four hours prior to measurements and had been resting in the supine position for 5 min before measurement and remained at rest (no movement) during measurement. There were no restrictions on voiding. Participants remained clothed with only hands and feet uncovered with the body positioned with the arms and legs abducted at a 30-45° angle from the trunk.

### 2.4. BIS Measurements

BIS measurements were performed with a Xitron 4200, HYDRA BIS device (Xitron Technologies, San Diego, CA, USA), which measures the impedance at 50 frequencies in the range from 5 to 1000 kHz. All measurements were performed in accordance with and earlier published paper [20].

Briefly, skin surface Ag-AgCl ECG-style gel electrodes were placed at wrists and ankle for whole-body measurements, and the skin was cleaned with alcohol (ethanol 75%) before the placement of electrodes. Measurements were performed with participants lying supine on a nonconductive surface (hospital bed/examination table) and abducted arms and legs. Intertwining of cables and similar sources of electrical interference was avoided.

Calibration of the device was tested every week with an electronic verification module (TS4201), supplied by the manufacturer (Xitron Technologies, San Diego, CA, USA).

All measurements were made at room temperature (21° to 25°C), between 08:30 and 15:30, by the same trained operator, and performed in triplicate with electrodes remaining in place between measurements. The total mean measurement time was 7 min, covering patient preparation and BIS measurements.

### 2.5. BIS Data Analysis

The quality of the impedance data was checked by visual inspection and statistical analysis of the Cole plots, using the ImpediMed SFB7 Multi-Frequency Analysis software (Version 5.4.0.3, Brisbane QLD, Australia) as described previously [22].

The precision of triplicate measurements made in each NS patient was based on the electrical parameters R_E_ and R_I_ [15, 18] and expressed by the coefficient of variation in percent (CV% = SD/mean *∙* 100%).

The average CV% showed to be low for R_E_ (0.5%) and R_I_ (1.5%) in the ANS patients (n = 8 triplicate measurements) and for R_E_ (0.2%) and R_I_ (0.4%) in the NSR patients (n = 5 triplicate measurements).

Resistance varies with the length and cross-sectional area of the conductor. For current passing through a body, neither path length nor cross-sectional area is well-defined. However, a resistance index (RI) can be calculated, which takes body size into account and is roughly proportional to the water volume [15]:

(1) $$\begin{array}{c} RI=\frac{H^{2}}{R} \end{array}$$

H is height and R is the relevant resistance. RI_E_, RI_I_, and RI_INF_ refer to the resistance indices for ECW (resistance R_E_), ICW (resistance R_I_), and TBW (resistance R_INF_), respectively.

### 2.6. Anthropometry

Weight and height were measured by trained personnel before impedance measurements were performed. Weight was measured on digital scales, with light clothes to within 0.1 kg. Height was measured without shoes, to the nearest 0.5 cm using a stadiometer. All measurements were made in duplicate with mean values used.

### 2.7. Biochemistry and Blood Pressure

Resting venous blood samples and blood pressures (Carescape V100 Monitor, GE Healthcare, USA) were collected in the ANS patients before commencing medical treatment. Blood chemistry was performed by trained biomedical laboratory scientists in an accredited hospital laboratory.

### 2.8. Statistics

Results were presented as mean ± standard deviation (SD), after test for normality, using Q-Q plots and statistical tests (Shapiro-Wilk and Kolmogorov-Smirnow) [23].

A paired two-tailed Student's t-test was applied to determine differences in impedance data between patients with active NS and at remission (ANS*∗* vs NSR). An unpaired two-tailed t-test was used to compare impedance data between patients with active NS with controls (ANS vs HC) and at remission with controls (NSR vs HC).

Statistical significance was set at a* p*-value < 0.05.

All statistical tests and graphical illustrations were prepared using the statistical software MedCalc ® (Version 17.9.7, Medcalc Software, Ostend, Belgium).

## 3. Results

### 3.1. Patient Characteristics

Table 2 summarizes the characteristics of all the subjects enrolled. When comparing the body weight of the groups, no significant changes were observed (*p* > 0.05).

Table 3 details the clinical data for the patients. All patients had normal or near normal renal function, and four of eight patients were hypertensive at admission.

Impedance measurements were only possible in five patients during remission because of repeated relapse in two of the patients and transition to another hospital of one patient.

**Figure 2:**
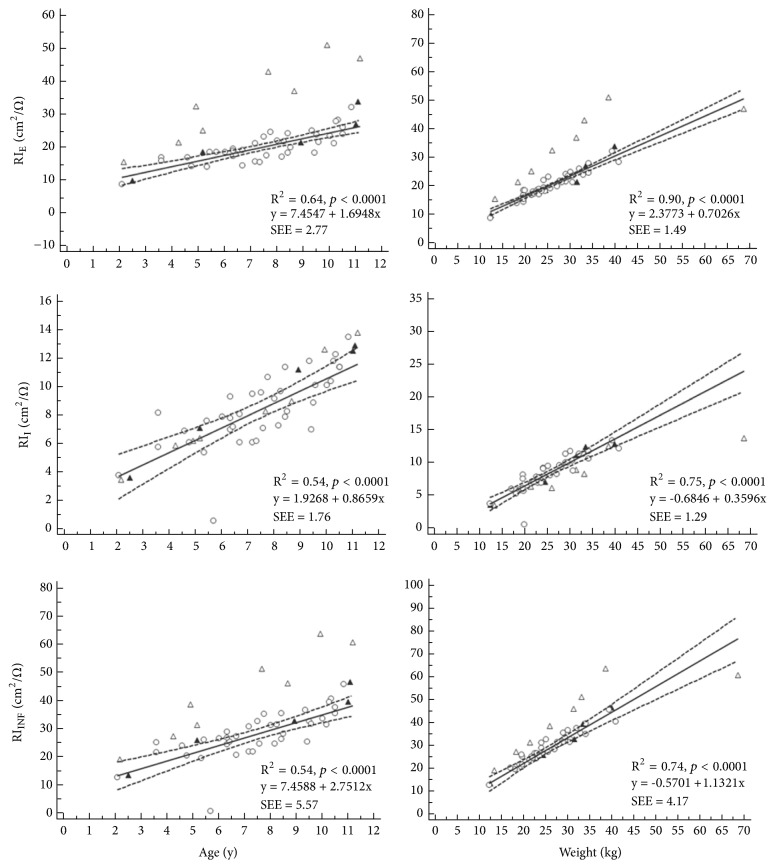
Relationship between resistance indices and age (left panel) and weight (right panel); ∆: ANS, ▲: NSR, and ○: HC, see Table 1 for group descriptions; dashed lines are 95% confidence intervals for regression lines based on the HC data.

**Figure 3:**
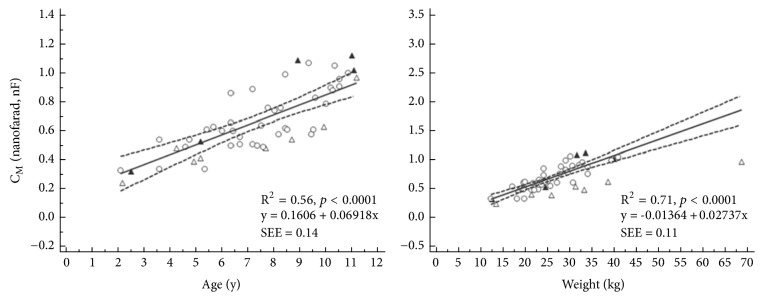
Relationship between cell membrane capacitance (C_M_) and age (left panel) and weight (right panel); ∆: ANS, ▲: NSR, and ○: HC, see Table 1 for group descriptions; dashed lines are 95% confidence intervals for regression lines based on the HC data.

One ANS patient was notable for being an apparent outlier for weight (Figure 2, right panel, and Figure 3, right panel); this patient was at the 100-percentile of weight for his age, using data from the ‘WHO child growth standards' [24].

### 3.2. Bioimpedance Parameters and Indices

Data are presented in Table 4 and corresponding* p*-values in Table 5. Resistance indices are presented as a function of age and weight in Figure 2. Cell membrane capacitance is likewise presented in Figure 3. A summary of the details is given in the following.

Overall, the results showed no significant differences between the children in submission (NSR group) compared to the healthy control children (HC group). Regarding ICW, the corresponding BIS parameter (R_I_) and resistance index (RI_I_) showed no significant differences between sick children (ANS group and ANS*∗* subgroup) and healthy controls (HC). See Table 5.

The mean values of R_E_ and R_INF_ were significantly lower for the sick children (ANS and ANS*∗*) than for the children in remission (NSR) or being healthy (HC). In numbers, the difference between ANS and HC groups was 369.2 Ω (-45%) for R_E_ and -212.6 (-37%) for R_INF_ (Table 4).

Correspondingly, the resistance indices RI_E_ and RI_INF_ (but not RI_I_) were significantly higher for the sick children than those in remission or being healthy (ANS*∗* vs NSR and ANS vs HC, respectively). In numbers, the difference between ANS and HC groups was +14.9 cm^2^/Ω (+75%) for RI_E_ and +14.7 cm^2^/Ω (+51%) for RI_INF_ (Table 4). These elevated values of RI_E_ and RI_INF_ (but not RI_I_) are graphically visible in both panels of Figure 2.

Cell membrane capacitance (C_M_) was also found to be significantly lower for the sick children (ANS and ANS*∗*) than for the children in remission (NSR) or healthy (HC). The graphical plots (Figure 3) reveal that only some of the individual ANS patients had lower C_M_ than the HC children.

## 4. Discussion

To the authors' knowledge, this paper demonstrated for the first time the relation between changes in disease status and cell membrane capacitance in paediatric patients with NS. These results were obtained using impedance spectroscopy, which allows the calculation of resistance at the optimal frequencies for ECW (*f* = 0) and TBW (*f* = ∞).

In the present study, BIS clearly indicated a higher ECW in the ANS patients compared to both the controls (HC group) and patients at remission (NSR group). Importantly, these findings accord with observations made in previous studies in NS patients [4, 25], where absolute fluid volumes were estimated using predictive methods.

As described in the Introduction, the use of BIS-modelled resistances avoids the assumptions inherent in derived equations for predicting fluid volumes. R_E_ represents the resistance of the ECW alone [26–29] while R_E_ and R_I_ have been used as indices of changes in ECW and ICW in neonates [11]. R_E_ and R_I_ are furthermore well-accepted parameters for characterizing material and biological properties, e.g., cell size, density, and the constituents of the extracellular and intracellular matrix [13, 30]. In our study, significantly lower R_E_ values were observed in the ANS patients compared to the HC. However, R_I_ values were the same as those in the healthy control cohort, indicating that the size of the intracellular water compartment is unchanged in the acute phase of NS, while the extracellular compartment alone is affected by the disease.

In contrast to the resistance values, resistance indices (RIs) standardize the values according to the subject's height. RIs have been shown to be an accurate reflection of absolute water volumes in children [31–33]. For the ANS patients, we observed that RI_E_ and RI_INF_ values were higher compared to the HC, and comparable with the HC for RI_I_. Again, it appears that only extracellular, not the intracellular, compartment is affected.

Consideration of basic BIS parameters can include electrical parameters other than resistance. Specifically, charge differences over cell membranes results in a capacitive effect [5]. In whole-body impedance analysis, cell membrane capacitance (C_M_) is a parameter measured at body level and represents an average of all membranes and tissue interfaces along the conductive path. Through cell death or cell destruction, the cell membrane loses its high capacitive properties [5], and upon general cell death, ECW expands and C_M_ decreases [5].

Even though there is no reason to suspect cell death or cell destruction in connection with NS, our results indicates that the cell membrane can be affected by the disease, despite the fact that ICW seems unaffected. This may indicate that there are components of the disease that affect the membrane negatively, e.g., inflammation and immune system activation [34]. Intriguingly, the pathogenesis of NS has previously been explained by a loss of negative charge in the glomerular basement membrane leading to heavy proteinuria. It is, however, unlikely that such changes would be discernible in a measurement of whole-body C_M_. This raises the possibility that these alterations in cell membrane function extend beyond the glomerular [35], but that possibility remains to be investigated more fully. Significant (*p* < 0.001) changes in C_M_ compared to healthy controls have also been observed in children suffering from sickle cell disease (SCD) [36].

### 4.1. Limitations

Some limitations to the study should be acknowledged. Only a small number of patients were available for study. This is a consequence of the low incidence of the disease with only around 2 new cases per 100,000 [37]. This carries the risk that the studied patients were not representative of NS patients in general; this is, however, considered unlikely since they exhibit the comparable clinical characteristics to other patients in the clinic. Even though significant differences between the sexes in body composition even when adjusted by height have been found in studies in healthy children [38], our sample was not large enough to conduct such an analysis.

Impedance measurements were performed as a wrist-ankle (whole-body) analysis. This arrangement does not make it possible to determine how the individual segments or discrete regions of the body contribute to the overall impedance with respect to NS, i.e., to identify in which of the segments/part where the greatest impedance changes occur. To obtain such information, it is necessary to perform focal or segmental measurements [15]. Normative data for segmental BIS in children aged 1-18 years are available for comparison [39], but it is unknown whether such an approach would be of value in NS patients.

## 5. Conclusion

This study shows how simple resistance indices could be used to assess changes in disease status in children with NS. This approach avoids invoking assumptions underpinning prediction of absolute volumes. More widely, such indices may prove to be useful screening tools for detection of fluid compartment imbalance in a clinical setting where the volume status of at-risk patients needs to be evaluated. In addition, it was observed that changes in C_M_ reflect disease status, suggesting that the capacitive properties of the cell membranes are affected by the disease. For further understanding of C_M_ in NS patients, studies are required with data specific for the different stages of NS that can be correlated with tissue pathology and, e.g., sex-specific differences.

## Figures and Tables

**Table 1:** Abbreviations and concepts used in this paper.

Abbreviation	Description
*General:*	
NS	Nephrotic syndrome
SFBIA	Single-frequency bioimpedance analysis
BIS	Bioelectric impedance spectroscopy (many frequencies)
*Study groups:*	
ANS	Children with active NS (n = 8)
ANS*∗*	Subgroup of ANS, same children as NSR group (n = 5)
NSR	Children from ANS group re-studied at the time of NS remission (n = 5)
HC	Healthy control children (n = 38)
*Physiological parameters:*	
ECW	Extracellular water (L)
ICW	Intracellular water (L)
TBW	Total body water (TBW = ECW + ICW)
*Impedance:*	
Z	Impedance for alternating current (Ohm, Ω).
R	Resistance (Ω)
*BIS parameters:*	
R_E_	R of the extracellular water (ECW)
R_I_	R of the intracellular water (ICW)
R_INF_	R of the total body water (TBW, measured at infinite frequency)
C_M_	Cell membrane capacitance (nanofarad, nF)
*Resistance indices:*	
RI_E_	RI = H^2^/R (cm^2^/Ω) of the ECW
RI_I_	RI of the ICW
RI_INF_	RI of the TBW

**Table 2:** Characteristics of the subjects enrolled in the study.

Parameter	ANS	ANS*∗*	NSR	HC
Sex (M/F)	7/1	4/1	4/1	23/15
Age (years)	6.9 ± 3.1	6.8 ± 3.1	7.7 ± 3.8	7.5 ± 2.2
Study weight (kg)	31.3 ± 17.1	28.5 ± 9.6	28.3 ± 10.4	25.3 ± 6.2
Height (cm)	120.7 ± 21.1	120.3 ± 21.8	126.1 ± 26.2	126.4 ± 14.2
BMI (kg/m^2^)	20.1 ± 4.6	19.1 ± 1.5	17.3 ± 2.0	15.6 ± 1.2

Data are means ± SD; for group abbreviations, see Table 1; BMI: body mass index.

**Table 3:** Clinical data for the ANS patients.

Parameters / Patients	1	2*∗*	3*∗*	4*∗*	5*∗*	6*∗*	7	8
Sex	M	F	M	M	M	M	M	M
Age (years, months)	5,2	7, 8	4,11	2,2	8,8	9,11	11,2	4,3
Blood pressure (mmHg)	109/71	130/93	109/68	110/70	116/77	133/73	109/56	88/62
Hypertension^†^ (yes/no)	yes	yes	no	yes	no	yes	no	no
Pulse (bpm)	155	77	92	109	104	99	102	97
P-Albumin (g/L)	7	9	9	10	9	9	4	10
P-sodium (mmol/L)	131	132	137	139	129	137	127	141
P-potassium (mmol/L)	3.5	4.9	5.5	3.5	5.0	3.8	3.9	3.6
P-creatinine (*μ*mol/L)	21	71	24	18	50	56	50	30
eGFR^‡^ (ml/min/1.73m^2^)	195	65	167	177	94	94	108	123
B-hemoglobin (mmol/L)	8.5	9.3	8.1	8.5	9.4	8.7	10.6	7.9

*∗*Patients in the subgroup ANS*∗*, which were restudied at remission, see Table 1.

^†^Hypertension defined as blood pressure above the 95% percentile for high and gender; ^‡^eGFR estimated by the Schwartz formula [19]. Reference ranges: P-Albumin (37-48), P-Sodium (137-145), P-potassium (3.5-4.6), and B-hemoglobin (6.5-8.9).

**Table 4:** Measured resistances (R), corresponding resistance indices (RI), and cell membrane capacitances (C_M_) in the study.

Parameters	ANS	ANS*∗*	NSR	HC
R_E_ (Ω)	447.1 ± 48.9	420.2 ± 43.5	752.6 ± 71.7	816.3 ± 73.8
R_I_ (Ω)	1871.6 ± 182.3	1919.7 ± 176.2	1799.5 ± 239.5	1922.3 ± 224.0
R_INF_ (Ω)	359.7 ± 33.3	344.1 ± 31.6	528.6 ± 46.0	572.3 ± 53.2
RI_E_ (cm^2^/Ω)	34.9 ± 11.7	37.2 ± 11.4	22.1 ± 8.4	20.0 ± 4.7
RI_I_ (cm^2^/Ω)	8.4 ± 3.3	8.3 ± 3.0	9.5 ± 3.7	8.6 ± 2.2
RI_INF_ (cm^2^/Ω)	43.3 ± 14.8	45.5 ± 14.3	31.6 ± 11.6	28.6 ± 6.9
C_M_ (nF)	0.53 ± 0.21	0.47 ± 0.13	0.82 ± 0.34	0.68 ± 0.20

Data are means ± SD; for group and parameter descriptions, see Table 1.

**Table 5:** Statistical comparison of results from Table 4.

Parameters	ANS*∗* vs NSR (paired)	ANS vs HC (unpaired)	NSR vs HC (unpaired)
R_E_ (Ω)	0.0006	<0.0001	ns
R_I_ (Ω)	ns	ns	ns
R_INF_ (Ω)	0.0006	<0.0001	ns
RI_E_ (cm^2^/Ω)	0.0270	0.0183	ns
RI_I_ (cm^2^/Ω)	ns	ns	ns
RI_INF_ (cm^2^/Ω)	0.0028	0.0402	ns
C_M_ (nF)	0.0307	0.0479	ns

For group and impedance descriptions, see Table 1. A Student's t-test was used to determine differences between the groups. Nonsignificance (*p* > 0.05) is denoted by “ns”.

## Data Availability

The data used to support the findings of this study are available from the corresponding author upon request.
